# Autophagy as a Survival Strategy for Eukaryotic Microbes Living in the Phyllosphere

**DOI:** 10.3389/fpls.2022.867486

**Published:** 2022-03-25

**Authors:** Kosuke Shiraishi, Yasuyoshi Sakai

**Affiliations:** Division of Applied Life Sciences, Graduate School of Agriculture, Kyoto University, Kyoto, Japan

**Keywords:** methylotrophic yeast, autophagy, pexophagy, Cvt pathway, phyllosphere, environmental adaptation, nutrient utilization

## Abstract

Autophagy is an intracellular degradation process that is highly conserved among eukaryotes at the molecular level. The process was originally revealed in the budding yeast, but the physiological role of autophagy in yeast cells had remained unknown as autophagy-deficient yeast mutants did now show a clear growth phenotype in laboratory conditions. In this review, we introduce the role of autophagy in the methylotrophic yeast *Candida boidinii* grown on the leaf surface of *Arabidopsis thaliana*. Autophagy is shown to be required for proliferation in the phyllosphere, and selective autophagic pathways such as pexophagy and cytoplasm-to-vacuole targeting (Cvt) pathway are strictly regulated during both the daily cycle and the host plant life cycle. This review describes our current understanding of the role of autophagy as a survival strategy for phyllosphere fungi. Critical functions of autophagy for pathogen invasions are also discussed.

## Introduction

Autophagy, a conserved mechanism of eukaryotes from yeasts to humans, is the major machinery for degrading the cytoplasmic compartments. During this process, the target cargo is sequestered into a double-membrane organelle called autophagosome and then transferred to the vacuole/lysosome (Suzuki et al., 2001; Suzuki and Ohsumi, 2007). Depending on the cargo, autophagy can be selective or non-selective. In non-selective bulk autophagy (hereafter referred to as “autophagy”), massive portions of the cytoplasm are sequestered by the autophagosome for degradation. This type of autophagy is frequently observed when nutrient levels are low, such as during starvation, and plays a critical role in the recycling of amino acids (Takeshige et al., 1992; Kim and Lee, 2014). In contrast, selective autophagy contributes to maintaining the number and integrity of organelles, as well as for protecting the cell from pathogen invasions. Selected cargo includes peroxisomes (pexophagy), mitochondria (mitophagy), endoplasmic reticulum (ER; ERphagy), ribosomes (ribophagy), and aggregates (aggrephagy) (Kirkin and Rogov, 2019). More than 40 autophagy-related (*ATG*) genes have been identified in the budding yeast *Saccharomyces cerevisiae* and other fungi since 1992, and their molecular mechanism, as well as various physiological functions, has been elucidated (Reggiori and Klionsky, 2013; Nakatogawa and Mochida, 2015; Oku and Sakai, 2016; Wen and Klionsky, 2016).

Of our particular interest is the role of autophagy as a survival strategy in microbes that inhabit the plant leaf surface. These microbes are exposed to various environmental stresses, such as extreme temperatures, ultraviolet radiation, drafts, osmotic pressure, reactive oxygen species, and low nutrients and must therefore adapt to these challenging conditions. While many studies conducted at the laboratory level have implied possible functions of autophagy for microbial survival in the phyllosphere environment, none of them have accumulated critical evidence for such implication until recently. This is because no experimental protocol has been established to monitor the direct response of microbes under natural conditions. To address this, we have developed a unique method in which cells of the methylotrophic yeast *Candida boidinii* are inoculated onto the leaf surface of the model plant *Arabidopsis thaliana*. The method allows us to observe the dynamics of yeast cells in the phyllosphere (Kawaguchi et al., 2011).

Methylotrophs are a diverse group of microorganisms that use reduced one-carbon (C1) compounds such as methane and methanol as a single carbon and energy source. Prokaryotic methylotrophs are capable of growth on a variety of C1 compounds, including methane, methanol, and methylamine, whereas the eukaryotic methylotrophs are limited to methanol utilization (van der Klei et al., 2006). After the first isolation in 1969 (Ogata et al., 1969), the eukaryotic methylotroph *C. boidinii* has been often isolated from plant-associated materials including the leaves (Kurtzman et al., 2011).

In the last two decades, the association and symbiotic relationship between plants and methylotrophs have become a critical research area (Ivanova et al., 2000, 2001; Omer et al., 2004). Among the parts of the plant, phyllosphere is receiving growing attention from scientific communities as the site of symbiosis for methylotrophic bacteria, in particular methanol-utilizing microbes. It was triggered by a report noting that some plants emit methanol from their leaves and provide microbes with habitats (Nemecek-Marshall et al., 1995), and was followed by a few critical articles including the one that estimated the global leaf area being 10^9^ km^2^ on which a total of approximately 10^26^ microbes exist (Lindow and Brandl, 2003), and another one that elucidated a symbiotic effect of *Methylobacterium extorquens* AM1 on plant leaves (Lidstrom and Chistoserdova, 2002). These reports prompted scientists to pioneer a new study area: phyllosphere microbiology. Today, we know that methylotrophs are universally distributed and are dominant on the plant leaf surface and that some of them have growth-promoting effects on the host plants (Vorholt, 2012).

Many phyllosphere microbes form spores either to survive under nutrient-limited conditions or to develop appressoria prior to invasion of the host plants, while asporogenous microbes also inhabit and survive for long periods on leaf surfaces over the entire life span of a plant without the aid of spores (Andrews and Harris, 2000; Jager et al., 2001; Ryder and Talbot, 2015). These asporogenous microbes must have developed certain survival strategies to adapt to the ever-changing phyllosphere environment. *C. boidinii* is one such plant-residing asporogenous methylotrophic yeast that can proliferate on growing plant leaves, assimilating methanol for its growth and survival (Kawaguchi et al., 2011). In addition to these characteristics, its biochemistry and cell biology are well studied, which has made the yeast *C. boidinii* the best model for investigating the role of autophagy as a survival strategy on the plant leaf surface. In this review, we summarize our recent studies and current understanding of the role of autophagy in phyllosphere microbes.

## Autophagy Is Required for Survival of Yeast on the Plant Leaf Surface

Our earlier studies revealed that the methylotrophic yeast *C. boidinii* could proliferate on the leaf surface of growing *A. thaliana* (Kawaguchi et al., 2011). Although the growth of another methylotrophic yeast *Komagataella phaffii* (synonymous *Pichia pastoris*) was also demonstrated using fluorescence microscopy, its cell growth could not be successfully monitored by quantitative PCR (qPCR) because it formed spores on plant leaves. Therefore, *C. boidinii* was chosen as a model for investigating the dynamics of phyllosphere microbes. Knock-out mutants deficient in the first reaction of methanol metabolism (alcohol oxidase: Aod1) and the peroxisome-assembly factor (Pex5) could not proliferate on *Arabidopsis* leaves, indicating that cells utilize methanol as a carbon source concomitant with peroxisome proliferation on plant leaves.

*CbATG1* encodes a pivotal kinase for all autophagic pathways and *CbATG8* encodes a marker protein of autophagic membranes (Matsuura et al., 1997; Kirisako et al., 1999). Yeast cells of these mutants and the wild-type strain were inoculated onto plant leaves and their growth was monitored. The number of the wild-type strain slowly increased three to four times in about 2 weeks, whereas that of *Cbatg1Δ* nor *Cbatg8Δ* strains did not, indicating that autophagy is required for yeast proliferation in the phyllosphere (Kawaguchi et al., 2011). Growth of autophagy-deficient yeast strains is almost unaffected by normal media used in the laboratory, whereas that of the methylotrophic yeast showed marked difference in its proliferation on the plant leaf surface. These results allowed us to reaffirm the critical role of autophagy in recycling nutrients under severe growth conditions.

**Figure 1:**
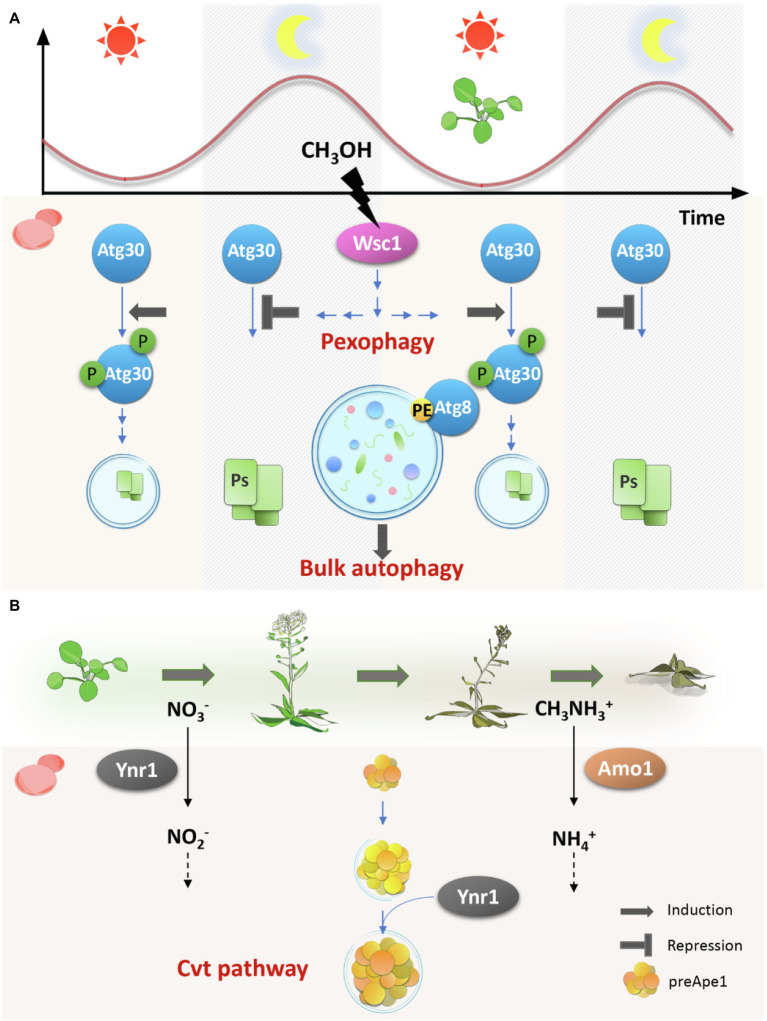
Schematic representation of the microbial regulation of autophagy in the ever-changing phyllosphere environment. Phyllosphere microbes make the best use of the autophagy mechanism to adapt to the host plant changes during both the daily cycle **(A)** and the host plant life cycle **(B)**. On young growing leaves, methanol (CH_3_OH) concentration is low in the light period and high in the dark and oscillates diurnally. As plants age, the available nitrogen source changes from nitrate (NO_3_^−^) to methylamine (CH_3_NH_3_^+^). To respond to these changes, phyllosphere microbes employ the autophagy mechanism. On plant leaves, bulk autophagy, chased by Atg8, is always active, whereas some selective autophagy pathways are tightly regulated. Pexophagy, monitored by Atg30, is negatively regulated in a Wsc1-dependent manner. Wsc1 senses the external methanol at the cell surface and transmits signals to the downstream factors. Only when the local methanol concentration is low, pexophagy is induced through the phosphorylation of Atg30. Cvt pathway, responsible for transporting vacuolar hydrolases like Ape1, which is synthesized as a precursor form (preApe1), is constitutively active in the phyllosphere. However, it recruits Ynr1, yeast nitrate reductase, only on aged leaves where methylamine is the major nitrogen source, and therefore, Amo1, but not Ynr1, is necessary. Ps: Peroxisomes, PE: Phosphatidylethanolamine, and P: Phosphorylation.

The autophagic activity was investigated with the yeast cells growing on plant leaves. The wild-type strain that expresses Venus-CbAtg8 (Cheong and Klionsky, 2008) was inoculated onto *Arabidopsis* leaves and collected the cell samples every 4 hours throughout the day. The light period was set for 16 h from 4 h to 20 h and the dark period lasted for 8 h from 20 h to 4 h. Autophagy was found to be induced throughout the daily dark–light cycle in *C. boidinii* cells (Figure 1A). Autophagic activity can also be examined by the modification of Atg8. During autophagy and autophagosome formation, Atg8 is lipidated by a ubiquitin-like conjugation system (Cheong and Klionsky, 2008). Immunoblot analysis showed the lapidated form of HA-tagged CbAtg8 throughout the daily cycle, which supports the autophagic activity detected by the cleaved Venus-Atg8 assay.

## Pexophagy Is Controlled Diurnally in the Phyllosphere

In addition to these conventional *ATG* genes, we speculated that pexophagy, i.e., a selective autophagy pathway that specifically degrades peroxisomes (Oku and Sakai, 2016), is required for yeast growth in the phyllosphere. This speculation came from our observations that *C. boidinii* uses methanol as a carbon source on the plant leaf surface and that the local methanol concentration oscillates dynamically during the daily light–dark cycle. For these observations, we established a yeast cell methanol sensor that shows fluorescence intensity corresponding to the environmental methanol concentration. The local methanol concentration on growing *Arabidopsis* leaves estimated by the sensor was higher in the dark period (20–4 h), and during early morning hours (4–8 h) but lower in the light period (8–20 h), corresponding to approximately 0–65 mM methanol (Figure 1A; Kawaguchi et al., 2011). In contrast, on wilted or died leaves, the methanol concentration exceeded 250 mM and did not oscillate.

Atg30 is known to be a pexophagy receptor and is required exclusively for peroxisome degradation (Farré et al., 2013). Similar to the *Cbatg1Δ* and *Cbatg8Δ* strains, the *Cbatg30Δ* strain did not proliferate on plant leaves, suggesting that pexophagy is essential. We then investigated the role of pexophagy by monitoring the peroxisome dynamics in yeast living on the plant leaf surface. Cells on growing *Arabidopsis* leaves contained one or two peroxisomal dots with some cytosolic fluorescence at 8 h in the light period. In contrast, at 0 h in the dark period, peroxisomal fluorescence increased with the simultaneous disappearance of cytosolic fluorescence (Kawaguchi et al., 2011). Together with the results on methanol fluctuation during the day (Figure 1A), these results indicated that peroxisomes are synthesized during the night and degraded in the morning. More specifically, pexophagy was suggested to be induced at around 8–12 h after the local methanol concentration dropped from the maximum to minimum.

The methylotrophic yeast best utilizes autophagy machinery and effectively recycles amino acids from peroxisomal proteins in the phyllosphere, where there is limited amount of nutrients available for microbes. A critical point of these findings is that only pexophagy is regulated throughout the daily light and dark cycle while non-selective bulk autophagy seems to occur all day. We consider that carbon and energy sources are stored in peroxisomes during the period of yeast survival on plant leaves and are degraded per requirement for recycling when the amount of obtainable nutrient sources decrease. Hence, peroxisome degradation is crucial for energy homeostasis in the phyllosphere environment. Several studies support our interpretation with the demonstration of the close association of peroxisomes with lipid droplets, which serve as energy storage modules in the form of neutral lipids such as triglycerides and sterol esters (Schrader, 2001; Binns et al., 2006; Valm et al., 2017).

## Wsc Family Proteins Sense the Extracellular Methanol and Negatively Regulate PEXOPHAGY

On the plant leaf surface, *C. boidinii* responds to fluctuation of the available methanol by controlling peroxisome synthesis and degradation. Our results on the changes in methanol-inducible gene expression that correspond to oscillation of the local methanol concentration indicated that the yeast regulates peroxisome synthesis at the gene expression level (Kawaguchi et al., 2011). The next question was to see how the yeast regulates peroxisome degradation on plant leaves where methanol concentration changes from 0 to about 65 mM. To address this question, we focused on the Wsc family proteins. They are known to be glycosylphosphatidylinositol (GPI)-anchored proteins, and detect and transmit cell wall status to the downstream factors as stress-sensors (Levin, 2005).

The growth test and transcript analysis with *K. phaffii* showed that KpWsc1 and KpWsc3 function as methanol-sensors during growth on methanol and that KpWsc1 responds to a lower range of methanol concentrations than KpWsc3, i.e., KpWsc1 for 3–15 mM and KpWsc3 for 30–150 mM (Ohsawa et al., 2017). Considering the methanol concentration in the phyllosphere, it was speculated that KpWsc1 plays a more important role than KpWsc3 in sensing the extracellular methanol and in the regulation of pexophagy on the plant leaf surface. Accordingly, pexophagic activity was monitored by chasing a YFP-tagged peroxisome protein, Pex11, between the wild-type and *Kpwsc1Δ* strains in the presence of methanol. The *Kpwsc1Δ* strain exhibited an earlier onset of pexophagy than the wild-type strain, suggesting that methanol sensing KpWsc1 works as a negative regulator and suppresses the degradation of methanol-induced peroxisomes (Ohsawa et al., 2021).

Phosphorylation of Atg30 is known to be coordinated with the interaction of Atg8 and Atg11 to induce pexophagosome formation at the peroxisomal surface (Farré et al., 2013). Atg11 is a required protein for most selective autophagy pathways, including pexophagy in yeast, and functions as a basic scaffold in assembling the specific phagophore assembly site (PAS) by interacting directly with the receptor, with itself and with several other proteins such as Atg1, Atg9, and Atg17 (Yorimitsu and Klionsky, 2005; Farré et al., 2013). To better observe the phosphorylation level of KpAtg30, the *Kpatg11Δ* strain was used as the background strain. Compared with the single *Kpatg11Δ* strain, HA-tagged KpAtg30 in the *Kpwsc1Δatg11Δ* strain was found to be more highly phosphorylated, which is consistent with the observed enhancement of pexophagy by the depletion of KpWsc1. With further investigations, we concluded that KpWsc1 and its downstream MAPK, KpMpk1, negatively regulate pexophagy in the presence of methanol concentrations higher than 48 mM (as summarized in Figure 1A; Ohsawa et al., 2021).

Our study showed that the local methanol concentration was the highest at 4–8 h and lowest at 8–12 h and that Atg30-dependent pexophagy was only observed at 8–12 h when the local methanol concentration dropped. In the plant leaf environment, pexophagy is supposed to be suppressed in a Wsc-dependent manner through dephosphorylation of Atg30 when the local methanol concentration is high. Phosphorylation of Atg30 is necessary for pexophagy even under non-selective autophagic conditions. From the Venus-Atg8 cleavage assay with yeast cells grown in the phyllosphere, we speculated that non-selective autophagy would occur continuously throughout the day due to nutrient limitation. So far, selective autophagy has been considered to be triggered by the phosphorylation of the receptor proteins such as Atg30 (positive regulation) for pexophagy (Farré and Subramani, 2016). Since peroxisome is necessary for methanol metabolism and must escape from pexophagy even under non-selective autophagic conditions, negative regulation of pexophagy is supposed to play a critical role in the phyllosphere survival strategy of microbes.

## Biosynthetic Cytoplasm-to-Vacuole Targeting Pathway Is Involved in Yeast Nitrogen Metabolism

In addition to the physiological significance of autophagy in carbon utilization, we investigated its role in nitrogen utilization using *C. boidinii* grown on *A. thaliana*. CbYnr1 encodes the yeast nitrate reductase, which catalyzes the conversion of nitrate to nitrite, whereas CbAmo1 encodes the peroxisomal amine oxidase, which converts methylamine to ammonia. Microscopic and qPCR analyses revealed that CbYnr1, but not CbAmo1, is necessary for yeast proliferation on young plant leaves (Shiraishi et al., 2015a). Inferring from the fact that yeasts survive on old leaves by using their highly developed peroxisomes as protein-storage organelles despite their inability to proliferate on them (Kawaguchi et al., 2011), their nitrogen metabolism was investigated during the host plant life cycle. The Venus reporter assay revealed that on growing young leaves, *CbYNR1*, but not *CbAMO1*, was expressed, while on wilting leaves *CbAMO1* expression was induced (Shiraishi et al., 2015a). These observations suggested that available nitrogen sources for *C. boidinii* change from nitrate to methylamine during plant life span. To the best of our knowledge, these findings represent the first description on the change of plant leaf environment with respect to the availability of nutrients for microbes.

Next, we explored the localization of CbYnr1 during plant aging and found that Venus-CbYnr1 was expressed in the cytosol in the cells surviving on growing leaves, indicating that CbYnr1 is utilized for the conversion of nitrate to nitrite. In contrast, on wilting leaves, Venus-CbYnr1 was found to form puncta, which urged us to hypothesize that CbYnr1 becomes unnecessary and is subjected to autophagic degradation induced by the adaptation to the change of leaf environment.

The intracellular dynamics of CbYnr1 was further investigated *in vitro*. In response to the nitrogen source shift from nitrate to methylamine, Venus-CbYnr1 formed dot-like structures that were detectable for 3 h. Subsequently, the Venus fluorescence was diffused inside the vacuole 12 h after the shift. In *Cbatg1Δ* cells, in contrast, Venus-CbYnr1 was not transported to the vacuole. The subcellular localization of CbYnr1 was also monitored in *Cbatg11Δ* and *Cbatg17Δ* cells. Atg17, together with Atg29 and Atg31, serves as a scaffold for autophagosome biogenesis and is required for autophagy (Ragusa et al., 2012). The frequency of Venus-CbYnr1 dot formation was low in *Cbatg11Δ* cells, but normal in *Cbatg17Δ* cells, suggesting that trafficking of Venus-CbYnr1 to the vacuole depends on selective autophagy but not on non-selective autophagy. Immunoblot assays supported the microscopic analyses. In *Cbatg11Δ* cells, band intensity of the cleaved form of Venus was clearly weaker than that in wild-type cells after the medium was shifted from nitrate to methylamine (Shiraishi et al., 2015a). Further investigations found that CbYnr1 transport to the vacuole occurred even during coexistence with nitrate after the medium shift (Shiraishi et al., 2015b).

Among the Atg11-dependent selective autophagy pathways (Suzuki, 2013), we focused on a constitutive biosynthetic pathway, cytoplasm-to-vacuole targeting (Cvt) pathway. It is responsible for the transport of several vacuolar enzymes, including aminopeptidase 1 (Ape1), aspartyl aminopeptidase (Ape4), and alpha mannosidase 1 (Ams1) (Scott et al., 1997; Hutchins and Klionsky, 2001). When the cells were shifted from nitrate to methylamine medium, mCherry-CbYnr1 often localized with Venus-CbApe1. We also observed co-localization of mCherry-CbYnr1 with CbApe4-Venus, another member of the Cvt complex. These results demonstrated that CbYnr1 forms dots associated with the Cvt complex and is sequestered into Cvt vesicles for ultimate delivery to the vacuole. Finally, localization of mCherry-CbYnr1 and Venus-CbApe1 was monitored in *C. boidinii* cells grown on plant leaves. Venus-CbApe1 dots co-localized with dot-like structures of mCherry-CbYnr1 on wilting plant leaves, but not on young leaves. Taken together, on young leaves, CbYnr1 was necessary for yeast proliferation and yeast utilized nitrate as nitrogen source, whereas on older leaves, methylamine metabolism was induced and CbYnr1 was subjected to degradation *via* the Cvt pathway (Figure 1B; Shiraishi et al., 2015a). These results proposed a new role for the Cvt pathway, which has been known to be a biosynthetic pathway in protein degradation. We speculate that inactive form of Ynr1 is recognized by some autophagic receptor protein and thereby recruited to Cvt vesicles for degradation. However, Atg19, a receptor protein of the Ape1 complex, is not found in *C. boidinii* genome. Thus, the detailed mechanisms, underlying the inactivated Ynr1 or other enzymes to Cvt vesicles, remain to be elucidated.

## Autophagy Plays a Critical Role for Pathogen Invasions

The importance of autophagy has also been reported in plant pathogenic microbes. A study showed that *Colletotrichum orbiculare*, a plant pathogen of melons and cucumber, dramatically decreased the infection efficiency to the host plant (Asakura et al., 2009) when it lost *CoATG8* or *CoATG26* genes. Atg26 is known to be an activator of pexophagy in *K. phaffii* and is preferentially required for the pexophagy process (Oku et al., 2003; Nazarko et al., 2007). Microscopic observation revealed that the *Coatg26* mutant developed appressoria but exhibited a specific defect in the subsequent host invasion step, implying a relationship between pexophagy and fungal phytopathogenicity. An interesting discovery was that the *Coatg8* mutant, which is defective in the entire autophagic pathway, could not form normal appressoria in the earlier steps of morphogenesis, while the *Coatg8* mutant was able to form them. CoAtg26-mediated pexophagy functions to recycle cellular components, and this might provide materials for the protein synthesis required for host invasion. These results indicated a specific function for Atg26-enhanced pexophagy during host invasion by *C. orbiculare*.

In addition to pexophagy, non-selective autophagy has been demonstrated as important for the establishment of rice blast disease in *Magnaporthe oryzae*. Genome-wide functional analysis revealed that 16 genes necessary for non-selective autophagy render *M. oryzae* unable to cause rice blast disease (Kershaw and Talbot, 2009). These are *MoATG1*, *MoATG2*, *MoATG3*, *MoATG4*, *MoATG5*, *MoATG6*, *MoATG7*, *MoATG8*, *MoATG9*, *MoATG10*, *MoATG11*, *MoATG12*, *MoATG13*, *MoATG15*, *MoATG16*, *MoATG17*, and *MoATG18*. Another study has elucidated that non-selective autophagy is required for nuclear degeneration in the spore, which is known to be essential for plant infection, during appressorium development (He et al., 2012). The importance of non-selective autophagy has also been disclosed with *Fusarium graminearum* by demonstrating that FgAtg20, a homologue to the sorting nexin ScAtg20 (Popelka et al., 2017), regulated morphogenesis and fungal pathogenicity (Lv et al., 2020).

Recently, the regulatory mechanism of pathogenicity-associated autophagy has started to be disclosed. In eukaryotic cells, histone modifications modulate DNA-related processes such as gene expression, DNA replication, and repair (Lawrence et al., 2016). In *M. oryzae*, histone acetyltransferase MoHat1 acetylated autophagy-related proteins MoAtg3 and MoAtg9 to orchestrate functional appressorium formation and pathogenicity. MoHat1 was found to be subject to regulation by the protein kinase MoGsk1 that modulated the translocation of MoHat1 from the nucleus to the cytoplasm with the assistance of MoSsb1, a protein chaperone (Yin et al., 2019). Another study revealed that MoSnt2 bound to promoters of autophagy genes *MoATG12*, *MoATG15*, *MoATG16*, and *MoATG22* to regulate their expression and that MoTor controlled *MoSNT2* expression to regulate MoTor signaling which lead to autophagy and rice infection (He et al., 2018).

Studies on the regulatory mechanism of pathogenicity-associated autophagy have extended to the signaling pathway and protein trafficking at the endosome. The ER stress was highly induced during *M. oryzae* infection and the cell wall integrity (CWI) MAP kinase kinase MoMkk1 was subject to phosphorylation regulation by MoAtg1. This study shed light on autophagy coordinating with CWI signaling to govern pathogenicity (Yin et al., 2020). The cargo-recognition complex MoVps35, MoVps26, and MoVps29 of the retromer, which is known to mediate protein trafficking through recycling cargo from endosomes to the trans-Golgi network in eukaryotes, is essential for appressorium-mediated host penetration by *M. oryzae*. The absence of the retromer function led to impaired biogenesis of autophagosomes due to the mislocalization of MoAtg8 to the vacuole (Zheng et al., 2015).

## Future Perspectives

Our studies have uncovered various aspects of the ever-changing phyllosphere environment. We found that the local methanol concentration of young *Arabidopsis* leaves fluctuates from 0 to approximately 65 mM during the day. When the plants age, wilt, or die, the methanol concentration exceeds 250 mM and stops oscillating (Kawaguchi et al., 2011).

In addition, we have also unraveled the survival strategy and mechanisms of the plant-residing asporogenous yeast *C. boidinii* on the phyllosphere environment. The methylotrophic yeast proliferates peroxisomes in the dark period and degrades them in the light period in accordance with the oscillating methanol concentration in the phyllosphere. Both peroxisome proliferation and pexophagy were found to be necessary for yeast growth on the plant leaf surface. With regard to nitrogen sources, nitrate is available for phyllosphere microbes on growing plant leaves, whereas methylamine becomes the major nitrogen source on aged plants (Shiraishi et al., 2015a). Multiple autophagic pathways have found to be controlled in a strict manner during the host plant life cycle as well. Ynr1, for example, escapes from pexophagy, which occurs during the light period on growing young leaves. However, it becomes a cargo member of the Cvt vesicle and is transported to the vacuole when the host plant ages (Kawaguchi et al., 2011; Shiraishi et al., 2015a). Such regulation of autophagy was suggested to exist in plant pathogenic fungi too, during differentiation of appressorium on plant leaves (Asakura et al., 2009). Therefore, regulation of selective autophagy plays a critical role in phyllosphere fungi. The findings presented here are important in that they provide insights on microbial adaptation mechanism and regulation of selective autophagy.

Not only proteins and organelles, but also bacteria and other intracellular pathogens have been found to be the target of selective autophagy (Nakagawa et al., 2004; Levine, 2005). Given that the phyllosphere environment is stressful with exposure to a variety of external stimuli including pathogens and that autophagy is considered as one of the major cellular processes for stress response, there must be many other important roles of selective autophagy that are yet to be discovered in the microorganisms that survive on plants.

## Author Contributions

KS and YS conceived and wrote the review. All authors contributed to the article and approved the submitted version.

## Funding

This research was supported by a Grant-in-Aid for Scientific Research on Innovative Areas (19H05709 to YS) from the Ministry of Education, Culture, Sports, Science and Technology, and by a Grant-in-Aid for Scientific Research (B) (19H02870 to YS).

## Conflict of Interest

The authors declare that the research was conducted in the absence of any commercial or financial relationships that could be construed as a potential conflict of interest.

## Publisher’s Note

All claims expressed in this article are solely those of the authors and do not necessarily represent those of their affiliated organizations, or those of the publisher, the editors and the reviewers. Any product that may be evaluated in this article, or claim that may be made by its manufacturer, is not guaranteed or endorsed by the publisher.
